# OpenCyto: An Open Source Infrastructure for Scalable, Robust, Reproducible, and Automated, End-to-End Flow Cytometry Data Analysis

**DOI:** 10.1371/journal.pcbi.1003806

**Published:** 2014-08-28

**Authors:** Greg Finak, Jacob Frelinger, Wenxin Jiang, Evan W. Newell, John Ramey, Mark M. Davis, Spyros A. Kalams, Stephen C. De Rosa, Raphael Gottardo

**Affiliations:** 1 Vaccine and Infectious Disease Division, Fred Hutchinson Cancer Research Center, Seattle, Washington, United States of America; 2 Agency for Science Technology and Research, Singapore Immunology Network, Singapore; 3 Department of Microbiology and Immunology, Stanford University, Stanford, California, United States of America; 4 Institute for Immunity, Transplantation and Infection, Stanford University, Stanford, California, United States of America; 5 The Howard Hughes Medical Institute, Stanford University, Stanford, California, United States of America; 6 Infectious Diseases Division, Department of Medicine, Vanderbilt University School of Medicine, Nashville, Tennessee, United States of America; 7 Department of Pathology, Microbiology, and Immunology, Vanderbilt University School of Medicine, Nashville, Tennessee, United States of America; 8 Department of Laboratory Medicine, University of Washington, Seattle, Washington, United States of America; 9 Department of Statistics, University of Washington, Seattle, Washington, United States of America; UCSD, United States of America

## Abstract

Flow cytometry is used increasingly in clinical research for cancer, immunology and vaccines. Technological advances in cytometry instrumentation are increasing the size and dimensionality of data sets, posing a challenge for traditional data management and analysis. Automated analysis methods, despite a general consensus of their importance to the future of the field, have been slow to gain widespread adoption. Here we present OpenCyto, a new BioConductor infrastructure and data analysis framework designed to lower the barrier of entry to automated flow data analysis algorithms by addressing key areas that we believe have held back wider adoption of automated approaches. OpenCyto supports *end-to-end* data analysis that is *robust and reproducible* while generating results that are *easy to interpret*. We have improved the existing, widely used *core* BioConductor flow cytometry infrastructure by allowing analysis to *scale* in a memory efficient manner to the large flow data sets that arise in clinical trials, and integrating *domain-specific knowledge* as part of the pipeline through the *hierarchical relationships among cell populations*. Pipelines are defined through a text-based *csv* file, limiting the need to write data-specific code, and are *data agnostic* to simplify *repetitive analysis* for core facilities. We demonstrate how to analyze two large cytometry data sets: an intracellular cytokine staining (ICS) data set from a published HIV vaccine trial focused on detecting rare, antigen-specific T-cell populations, where we identify a new subset of CD8 T-cells with a vaccine-regimen specific response that could not be identified through manual analysis, and a CyTOF T-cell phenotyping data set where a large staining panel and many cell populations are a challenge for traditional analysis. The substantial improvements to the *core* BioConductor flow cytometry packages give OpenCyto the potential for wide adoption. It can rapidly leverage new developments in computational cytometry and facilitate reproducible analysis in a unified environment.

This is a *PLOS Computational Biology* Software Article.

## Introduction

Technological advancements in cytometry instrumentation have enabled rapid, multidimensional quantification of millions of individual cells to define cellular subpopulations and assess cellular heterogeneity [1]–[6]. Traditional analysis of these data involves time-consuming sequential manual gating that is untenable for larger studies in the long-term [7]. The subjectivity of manual gating introduces variability into the data and significantly impacts the reproducibility, robustness and comparability of results, particularly in multi-center trials [7], [8]. Automated data analysis pipelines [9]–[14], which have developed rapidly in the past few years [12], have failed to gain widespread adoption outside of specialized computational labs. We hypothesize this is due to the usual factors that limit the uptake of new technologies, specifically a perceived difficulty in to learning to use the tools, and a lack of confidence in the veracity of generated results [15]. Although a recent study by the FlowCAP consortium aimed to boost user confidence in the viability of automated gating methods, many of the pipelines described therein were tailored for exploratory, discovery-oriented data analysis, which often generates tens to hundreds of cell population phenotypes, lacking the hierarchical cell population relationships that make the data easier to interpret [12]. Consequently, many of these tools are less suitable for use in a clinical research setting where analysis must be standardized, reproducible, and interpretable [16]–[18].

Clinical assays must be extremely well controlled in order to generate data that is comparable over time and across centers [8]. In order for automated approaches to gain traction in clinical flow studies, pipelines must produce results that are reproducible, robust, and easy to interpret. Likewise, the pipelines must be easier to use for flow data analysts who are not trained programmers, they must facilitate data sharing and collaboration, and they must enable users to make comparisons of different analysis approaches in order to evaluate the viability of an automated vs. a manual approach and thereby build user confidence. While the high-dimensional, unbiased automated gating approaches that have been developed to date have been shown to expedite the gating of FCM (flow cytometry) data sets and to remove the subjectivity intrinsic to manual gating [10]–[14], [19], these methods do not meet all of these other criteria. The output of high-dimensional gating methods generally requires post-processing and careful manual curation to ensure valid results [20], [21]. Yet, in clinical research, cell populations of interest are generally defined *a-priori*, and there is less immediate need for exploratory approaches. The implications for automated gating in a clinical trials setting are that any proposed analysis method must be validated and verified by demonstrating certain performance characteristics, including: accuracy, precision, reportable range of test results for the test system, verification that reference intervals are appropriate for the laboratory's patient population [22]. The specifics would vary from assay to assay, but robustness and reproducibility, i.e. the ability to consistently and accurately identify target populations, are key requirements that high-dimensional, unsupervised methods cannot yet meet.

In order to begin addressing the above issues, some high-dimensional automated gating tools have taken a supervised or semi-supervised approach to gating. One such tool is the Xcyt software, which aims to mitigate problems of population matching by implementing a supervised classification approach wherein the user fits a model to training data, which is then used to classify cells in other samples [19]. This facilitates population matching and helps ensure that consistent cell populations are identified across samples [20]. We believe this is a step in the right direction, but the approach is limited by an appropriate choice of template, and by the mixture modeling framework, which has known limitations [12]. Furthermore, constructing complex, multi-step analysis pipelines in that framework still requires extensive coding by the user. What is lacking in the computational flow ecosystem is a software infrastructure that provides the flexibility to quickly construct data analysis pipelines that can utilize different gating algorithms and handle large data sets efficiently. In our view, “gating” has become easier, while getting the data into and out of the different gating algorithms remains a difficult task. Without such infrastructure, there will continue to be a disconnect between the requirements of flow cytometry experimentalists, and the features provided by available tools [12].

In order to help bridge this gap, we have developed the OpenCyto framework. A recent review of flow cytometry bioinformatics highlighted the four components of an analysis pipeline: preprocessing, cell population identification, population matching and correlation with outcome variables [21]; OpenCyto fulfills all of these components, and aims to meet the challenges of *ease of use*, *interpretability*, *scalability*, *collaboration*, *comparative analysis*, *reproducibility and robustness*, while allowing analysts to integrate *domain-specific knowledge* into the analysis pipeline. We have extended the core BioConductor flow cytometry packages (*flowCore* and *flowViz*) to support HDF5/NetCDF-backed data storage via the new *ncdfFlow* package, and made the flow *visualization* framework more flexible and familiar to flow data analysts. This allows all FCM packages that utilize the core flow data structures in R to efficiently handle large data sets and benefit from improved visualizations. We have also developed two new packages; *flowWorkspace* implements the data structures required to represent hierarchical gating pipelines that can chain together different gating algorithms in series, allowing users to select the best suited analysis tools from BioConductor's flow cytometry ecosystem, or to import manually gated data from external tools like FlowJo (TreeStar Inc., Ashland, OR). The *openCyto* package abstracts the data, and simplifies construction of these pipelines via *gating templates* that don't rely on a training data set. These templates are staining panel specific, and provided experiments are well standardized, a template can be applied to any flow data set utilizing the same staining panel. The core FCM packages have exhibited a ten-fold increase in use over the past year (from 486 to 4776 distinct IP downloads in ten months), consequently this new infrastructure has the potential to have a significant impact for the computational flow community.

## Design and Implementation

### Overview

**Figure 1:**
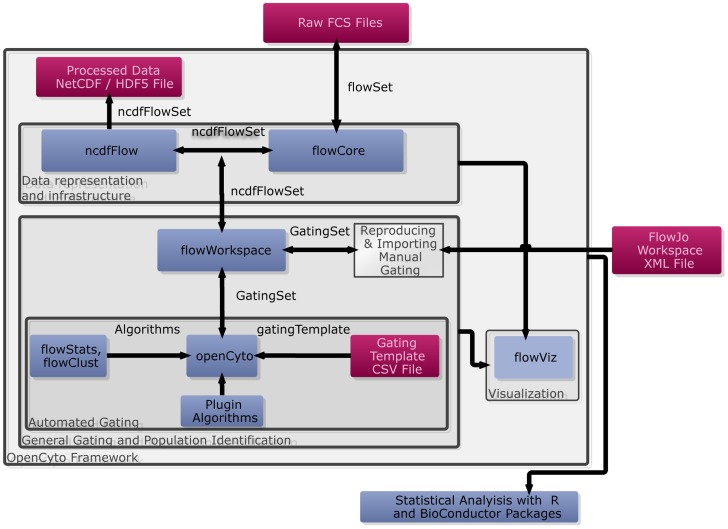
An overview of the OpenCyto infrastructure. When reproducing manual gating, raw FCS files and FlowJo workspace XML files are read into the R environment using *parseWorkspace*, creating a *GatingSet* object that represents the compensated, transformed and gated data stored in an *ncdfFlowSet* on disk. Cell populations annotated with gates can be visualized using *plotGate*, from the *flowViz* package Gating schemes can be visualized using *plot*. To perform automated gating, the user defines a *csv* representation of a gating tree, which is read by the *OpenCyto* package to generate a *gatingTemplate object*. This template can be applied to a *GatingSet* containing data, but no gates, provided the data uses the markers defined in the template. OpenCyto utilizes built-in automated gating methods, or external methods registered via a plug-in framework, to gate different cell subsets and populate the *GatingSet* with data-driven gate definitions for each sample. Manual and automated gating may be readily compared within a single framework. Cell populations and features can be extracted for further statistical analysis with other R and BioConductor software packages. Data (red boxes), software packages (blue boxes), framework functionality (gray boxes), and data flow/data structures (arrows/labeled arrows) are represented. *flowCore*, *flowStats*, and *flowViz*, are the *core* Bioconductor flow packages that benefit from the substantial infrastructure changes we have made to improve scalability and data visualization.

The OpenCyto framework is a collection of well-integrated open-source R/BioConductor packages: *ncdfFlow*, *flowCore*, *flowViz*, *flowWorkspace*, and *openCyto* (the package). The OpenCyto infrastructure and typical workflow is summarized in Figure 1. The framework consists of a near-complete re-implementation and extension of the *core* BioConductor flow cytometry infrastructure [23]–[26], allowing it to process large data sets (limited only by disk space and the maximum file size supported by the operating system) through native support of the HDF5/Network Common Data Format (NetCDF) [27]. The *flowWorkspace* package is built on top of this infrastructure and provides a new set of core objects termed *GatingHierarchy*, *GatingSet* and *GatingSetList*, which are used to associate an individual sample or set of samples with preprocessing (compensation and transformation) steps and *hierarchical* gating scheme(s).

The *openCyto package*, which depends on the *core infrastructure*, implements a *hierarchical automated gating pipeline* that incorporates data preprocessing and reproducible, data-driven automated gating. Installing the *openCyto* package will install all its dependencies, including the *core* flow cytometry packages. Throughout the paper, we use the name *OpenCyto* (capital O) to refer to both the package and the framework, and will make the distinction when necessary. The hierarchical structure encodes relationships amongst cell subpopulations that have a familiar interpretation and are informed by the biology of the study. Additionally, this structure allows effortless cell population matching since the relationships amongst cell sub-populations are preserved across samples (ensuring each sample has the same population defined). The objects representing the data analysis are associated with sample and experimental metadata, such as outcome variables, making it straightforward to leverage the classical statistical tools of the R language to test for association between extracted cell populations and study outcome within a single framework.

The hierarchical gating structure diverges from the usual approach to automated gating, wherein all cell events are clustered on all dimensions simultaneously, however, this structure encodes significant domain-specific knowledge about an experiment, including the relationships amongst known cell populations that can be defined using a given set of markers. In fact, users who run automated pipelines often impose such a structure implicitly, either as part of data cleaning prior to gating (equivalent to manual gating on debris or boundary events), or by applying automated algorithms in a sequential manner to subsets of flow data (e.g. lymphocytes are often gated prior to, and separate from other markers, even in a typical discovery-oriented automated analysis). OpenCyto provides a framework that forces these steps to be explicitly included and tracked, facilitating reproducibility. This framework can be used to encode analysis using high dimensional gating algorithms, or traditional sequential gating (Figure S1 A, B). The latter may be imported from a manual analysis using external software (i.e., FlowJo) or via an automated analysis wherein gates are defined in a data-driven fashion using the variety of gating algorithms available in R/BioConductor. A hybrid approach may also be used wherein high-dimensional gating can be applied to specific cell subpopulations defined using hierarchical 2-D gating (Figure S1 C). Importantly, new algorithms can be easily integrated via a plug-in architecture, ensuring the framework can adapt and remain current with new technological developments. The core *flowWorkspace* objects are implemented in C++ for increased speed and memory efficiency.

### OpenCyto Facilitates Comparative Data Analysis

The framework supports importing gates from external software (i.e., FlowJo), faithfully reproducing manual analysis within R. The gated data objects can be saved to disk. This allows users to easily share raw FCM data, together with associated analyses, and facilitates the comparison of automated or semi-automated gating approaches with manual gating, and enabling validation of automated gating schemes against expert manual results. Furthermore, these features facilitate collaboration between computational and non-computational researchers and have enabled the development of advanced downstream data analysis algorithms for FCM data in vaccine trials [28], [29], as well as a recent comprehensive comparison of automated gating algorithms via the FlowCAP effort [12]. The framework also facilitates extracting specific cell populations for downstream analysis from any step of a pipeline, as we demonstrate with the two data sets analyzed here [30].

### Automated Analysis via Gating Templates Promote Reproducible Results

The *OpenCyto* package allows users to define general gating schemes represented by *gatingTemplate* objects. A gating scheme is user-defined in a text file (CSV) that describes *cell sub-populations* and their *parent-child-relationships*, together with *markers* and *algorithms* to be used to gate each population. This defines a *tree* much like one would define a gating scheme in traditional manual analysis, except that the user does not draw gates or define gate coordinates. *OpenCyto* generates the gate coordinates in a data-driven manner when the template is applied to a data set. It can be applied to any data set that uses the staining panel defined in the template. This facilitates reproducible research by standardizing the data analysis as well as promoting code reuse. Given the template and the same data, any user will be able to generate the same results. This also simplifies repetitive data analysis for users that frequently analyze data from the same types of assays.

### OpenCyto Pipelines Are Flexible and Extensible

OpenCyto supports a number of different built-in *automated gating algorithms*, including high-dimensional model-based methods (*flowClust*) [31], density-based methods (*mindensity*, flowDensity), rare cell population identification (*tailgate*, quantileGate), and various specialized gate algorithms (*singletGate*, *transitional B-cell*, and *referenceGate*). These methods provide a suite of tools that are well suited to gating lymphocytes, transitional B-cells, singlets, bimodal or multimodal populations, or rare cell populations. They can be combined within a single gating scheme to generate an optimal gating strategy for a given staining panel. Additional algorithms are supported via a plug-in framework. The DNA vs. DNA gate used to analyze the CyTOF data set presented here, and the *flowDensity* algorithm used in FlowCAP III, are two such examples integrated into the OpenCyto framework [30] via the plugin mechanism.

### OpenCyto Will Promote Flow Standardization Efforts

A core flow laboratory will generally have a set of well-standardized flow assays with fixed staining panels. For example, a core lab may have a standard T-cell assay that always uses the same staining panel. The OpenCyto *GatingTemplate* is designed to take advantage of this. Our automated gating approach allows the gating of each cell population to be fine-tuned via cell sub-population specific parameters in the template definition in order to optimize cell population identification for the assay. OpenCyto is sufficiently robust that, once set up, the *GatingTemplate* is *reusable* for any data set from the same lab, provided the assay remains well standardized (*i.e.*, instrument parameters remain well-controlled and stains don't vary too much in their performance). OpenCyto promotes rapid, exhaustive, and most importantly, *reproducible* gating, and the results are easy to interpret in the context of a standard gating hierarchy. Importantly, variation due to differing technical expertise of data analysts can be eliminated [8].

## Results

In this section we describe the analysis of two data sets using the OpenCyto framework. The raw and processed data, as well as the R code used to generate the figures in this paper as well as further documentation, can all be found online at http://www.opencyto.org. The first data set is from the HIV Vaccine Trials Network (HVTN) consisting of FCS data from clinical trial HVTN080 [32] (http://flowrepository.org accession FR-FCM-ZZ7U). The data set is a 13-parameter intracellular cytokine-staining (ICS) assay comparing pre- and post-vaccine T-cell response to antigen stimulation (Env, Gag, Pol) and negative control stimulation (i.e. background) from 47 subjects consisting of 470 FCS files, 18.8 GB in size. The study compared two vaccine regimens, Pennvax B alone and Pennvax B + IL12 DNA. We gate these data with *OpenCyto* and show that the results recapitulate manual analysis. The second data set is smaller in size (74 MB), but higher dimensionality (32 parameter). It is mass cytometry time of flight (CyTOF) data from a study examining the diversity and combinatorial expression of nine cytokines and functional markers on CD8^+^ T cells. Here we re-analyze the three samples presented in the published figures of the original study [30].

### OpenCyto Can Recapitulate Manual Gates

The data in the original HVTN080 study was manually gated at the HVTN using FlowJo, and distributed amongst 16 workspaces. We imported the FlowJo workspaces and raw FCS files into R using the *flowWorkspace* package.

**Figure 2:**
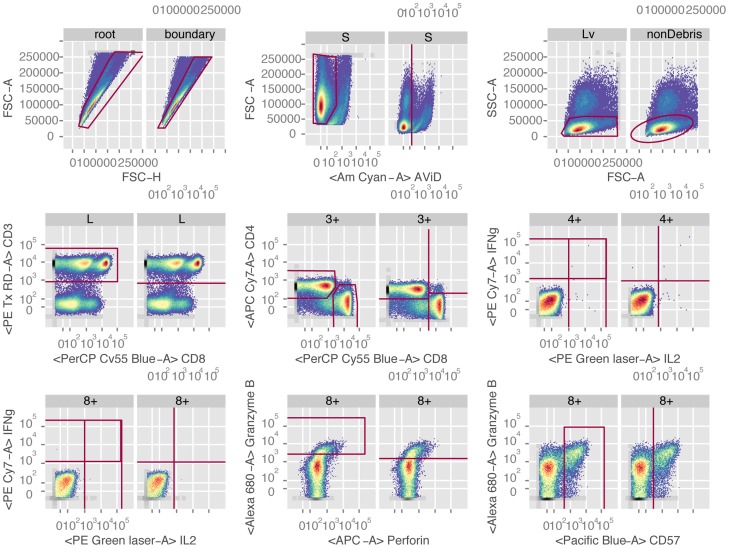
Comparison of a subset of manual gates and OpenCyto automated gates for a representative sample from the HVTN080 ICS data set. The automated gates are data-driven. Each panel shows a corresponding manual and automated gate side-by-side. The left panel is the manual gate; the right panel is the OpenCyto data-driven gate. Parent population names differ between manual and automated gates for singlets and lymphocytes because the automated gating hierarchy differs from the manual gating by including boundary and boundary debris gates, respectively, before these populations. Starting at the top left and proceeding along the rows, the gates shown are singlets, live cells, lymphocytes, CD3^+^ T-cells, CD4^+^ and CD8^+^ T-cells, IFN-γ^+^ and IL2^+^ expressing CD4^+^ and CD8^+^ T-cells, and Granzyme B^+^ and CD57^+^ expressing CD8^+^ T-cells. The manual and automated gates are very comparable.

To perform automated gating of this data, we defined a *gatingTemplate* (File S1) to reproduce the manual gating hierarchy (Figure S2 A, B) using the variety of automated gating algorithms available to the *openCyto* package. Briefly, the data were gated for CD4^+^ and CD8^+^ T-cells using the FSC vs. SSC (lymphocytes), Live/Dead, CD3, CD4, and CD8 markers, followed by gating of cytokine positive cells within the two T-cell subsets. The automated gating hierarchy has additional gates to remove boundary events and debris (Figure S2). A subset of automated manual gates from a representative sample are shown for comparison in Figure 2 (the complete gating scheme is shown in Figures S3 and S4). The manual and automated gates are very similar, and share a common hierarchical structure that facilitates direct comparison of cell populations between them. This is an important feature of OpenCyto, as it produces cell subsets that are easy to interpret in terms that are familiar to flow data analysts. The relationships amongst known cell populations are preserved in the gating hierarchy.

In order to better quantify the similarity of the cell subsets identified through manual and automated gating, we extracted the proportions of CD4^+^ and CD8^+^ T-cells in all 2^5^ disjoint cell subsets of the 5 functional markers (IFN-γ, IL-2, CD57, Granzyme B, and TNF-α) from the manual and automated gating results (stored as *GatingSet* objects). Although we are interested in comparing the cell subset proportions between manual and automated gating, not all of the 64 possible cell subsets are necessarily of interest. Importantly, an endpoint of this type of study would be to identify cytokine producing cell subsets where the proportion of cells increases significantly upon antigen stimulation at the post-vaccination time-point compared to the pre-vaccination time-point. To this end, and to filter out uninteresting subsets, we fit a linear mixed effects model (with random subject effect) to the background (negative control) corrected proportions of each cell subset and tested for a significant and positive interaction coefficient between visit and treatment (see Supporting Text S1, one-sided generalized linear hypothesis test, Bonferroni adjusted p-value≤0.05). We selected significant cell subsets from the model for further analysis. This ability to extract interesting features from flow cytometry data directly for downstream analysis within a rich statistical analysis environment like R, while maintaining access to the raw data is a powerful feature of OpenCyto that can help limit the propagation of data entry errors sometimes introduced when data are copied and pasted or annotated in external data analysis tools, and that promote the production of reproducible research results.

**Figure 3:**
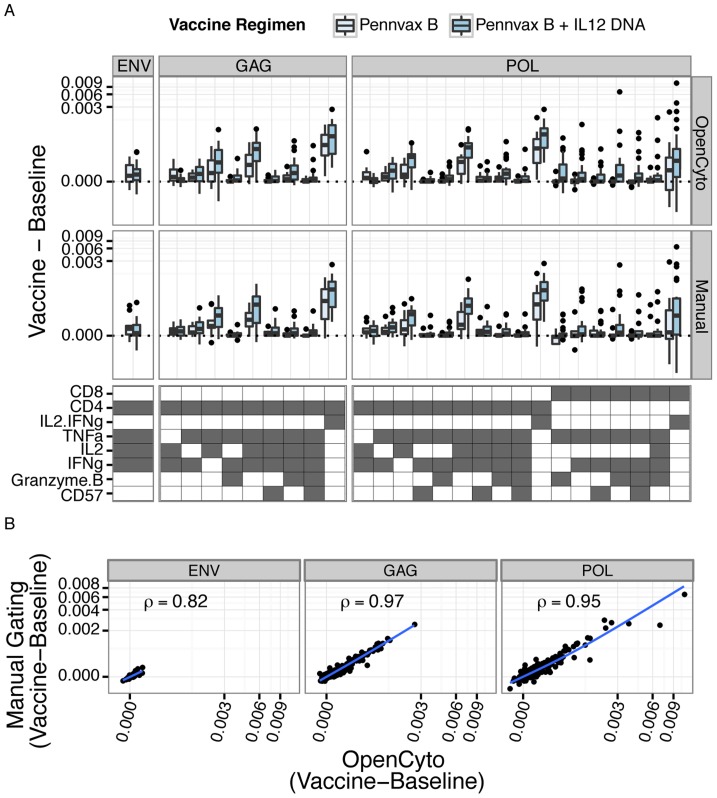
Comparison of OpenCyto automated gating and manual gating (performed with FlowJo and imported and reproduced in R using OpenCyto) for HVTN 080. A) Box-plots of the paired differences (post-vaccination – baseline) in proportions of cytokine-producing cells from significant cell subsets identified by the linear model (see Supplementary Methods) for each stimulation condition, gating method, and vaccine regimen. Differences between baseline and post-vaccination are background-corrected (stimulated – non-stimulated). There were no significant differences between the observed distributions for manual or OpenCyto gating (paired Wilcoxon test). B) Scatter plots comparing manual gating vs. OpenCyto gating. The per-subject, background-corrected difference between vaccine and baseline is plotted for OpenCyto and manual gating, with concordance correlation coefficients shown for all stimulations.

In Figure 3A, we show box-plots of the paired differences for cell subsets identified by the model, and stratified by vaccine regimen. We observe a vaccine-regimen specific response to antigen stimulation within the Gag and Pol treatment groups. The *Env* stimulation shows the weakest response, with the fewest significant cell subsets, followed by Gag, and Pol. Furthermore, the response in CD4^+^ T-cells is greater than in CD8^+^ T-cells, and the response following Pennvax B + IL12 DNA vaccination is greater than Pennvax B alone. The CD4^+^ and CD8^+^ T-cell subsets producing IFN-γ or IL-2 (IL2.IFNg) are used by the HVTN as the readouts for the ICS assay. We note that we detect an antigen-specific response in these subsets and that the CD4 subsets have the strongest response to antigen stimulation by both methods, consistent with the original study findings [32],[33]. Most importantly, there are no significant differences between the manual and OpenCyto gating results for any of the cell subsets (two-sided paired Wilcoxon test). The concordance correlation coefficient between manual and automated gating across all subsets was 0.82, 0.96, 0.97, respectively for Env, Gag, and Pol stimulation, further demonstrating that OpenCyto can faithfully reproduce manual gating results in an automated manner, even for rare cell populations (Figure 3B) [34], [35]. The ability to directly compare manual vs. automated gating in an objective and quantitative manner can help users to develop new gating templates for their assays while promoting confidence in the veracity of automated gating results.

An important feature of the HVTN ICS data presented here is that it is a highly standardized assay within the HVTN lab. This standardization highlights an important feature of our framework. We were able to construct and refine the OpenCyto *gating template* (Supplementary File 1) for this assay by working with just a few subjects' worth of data, rather than the entire data set. OpenCyto gating templates are staining-panel specific, but data agnostic, and can be applied to any standardized data set that uses the same staining panel. In this way, the *gatingTemplate object* abstracts the data, eliminating the need to write data set specific code. This functionality should be particularly attractive to *core facilities* and *clinical trials networks* that regularly process large numbers of samples through standardized flow cytometry assays. The analysis of such data is standardized, but time consuming; it is an important niche we have designed our framework to fill.

### OpenCyto Improves Gating of Markers with High Variability

**Figure 4:**
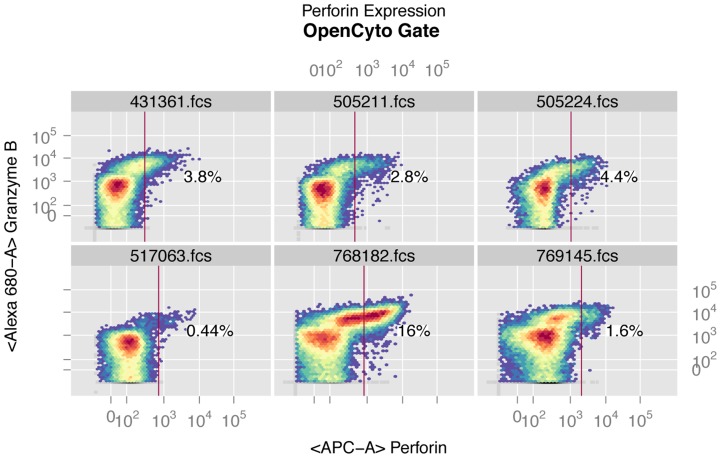
Example of OpenCyto automated gates on the perforin channel for CD8^+^ T-cells for six randomly selected samples from the HVTN 080 ICS data set. The perforin marker exhibits staining variability as evidenced by the varying width and position of the negative peak and was not gated by the manual template-gating approach. Despite this variability, OpenCyto data-driven automated gating is able to identify a reasonable threshold for perforin positive cells.

One of the markers (perforin) in the HVTN data set shows considerable variability in MFI that has been described elsewhere [29]. This marker was not included in the original analysis of the data [32]. In order to determine whether OpenCyto could correctly account for the sample-to-sample variation in this marker when placing data-driven gates on the perforin-positive cells, we included perforin in the pipeline. Existing approaches used to account for this variation include cell-subset and channel specific data normalization approaches [29]. Figure 4 shows OpenCyto gates for CD8^+^ T-cells expressing perforin from six randomly selected samples in the ICS data. Perforin staining shows clear variability both in the width and position of the negative peaks. Despite this variation, the automated gates are reasonably placed to discriminate perforin negative from perforin positive cells. As a proof of principle, automated gating of perforin allowed us to detect a vaccine regimen specific trend for post-vaccine response in CD8^+^ T-cells stimulated with Pol antigen, expressing *any* cytokine (i.e., IL2 or IFN-γ or TNF-α) and (i.e., simultaneously with) perforin in the Pennvax B + IL12 DNA group but not in the Pennvax B group alone (Figure S5). This trend was present, but not significant in CD4 T-cells, in agreement with the known biology of perforin expression (i.e., constitutive expression on CD8^+^ T-cells). The decision to model expression of *any* cytokine jointly with perforin is motivated by the fact that perforin is constitutively expressed on CD8 T-cells and interpretation of its expression in response to antigen stimulation is only valid when considered jointly with other cytokines. We examined the POL-1-PTEG stimulation because, for other T-cell subsets, it exhibited the strongest response of all the stimulations considered (Figure 3). Importantly, this analysis was only possible for the OpenCyto gated data since perforin was not gated in the manual analysis. Our automated gating approach can allow markers that exhibit such staining variability to be used regularly for downstream analysis, without requiring time-consuming manual intervention to adjust traditional template gates.

Until now, a limiting factor of the BioConductor FCM infrastructure has been the inability to handle large data sets. We have eliminated this shortcoming by implementing support for disk-backed storage of FCM data in HDF5/NetCDF [27], [36]–[38] files. The *flowSet* and *flowFrame* data structures, which represent FCS files and sets of FCS files (sharing a common set of markers), can now store their data on disk in a NetCDF-compatible file (using the HDF5 library), which is efficiently accessed by slices (each slice represents an FCS file), eliminating the limitations of storing an entire flow study in memory. We used this functionality to analyze the HVTN ICS data set. We were able to load and merge the 470 FCS files and corresponding manual gates from the 16 FlowJo workspaces (corresponding to 16 plates) within a single R-session, and manipulate and interact with the data. To our knowledge, no other automated flow data analysis infrastructure allows for this kind of scalability for event-level data (we note that cloud-based platforms like Cytobank [18] scale well, but do not currently handle *automated gating*). Since large, manually gated data sets are often stored across multiple *workspaces*, this functionality is critical for automated analysis of the data sets generated in clinical research.

Importantly, the time required to perform automated gating using OpenCyto can be greatly reduced compared to manual analysis, although it is dependent on the dimensionality of the data set. For the ICS data set, the majority of the computation time is spent gating the individual samples, whereas for the CyTOF data set (described next), most of the time is spent computing the Boolean subsets (Table 1). The time to extract Boolean gates in OpenCyto is already an improvement over some manual analysis tools (7.4 minutes for the ICS and 2.6 minutes for the CyTOF data). This improvement is attained through an optimized polyfunctionality gating method that caches event indices for each gate, ensuring that cell subset counts are returned for each cell subset in an efficient manner. Although there is some overhead in retrieving data from the NetCDF/HDF5 file, the benefits of being able to access single-cell data from an entire study at once outweighs the additional cost in time. For smaller studies, if sufficient RAM is available, storage of FCS data in *flowSets* is still an option.

**Table 1:** Performance metric of OpenCyto on the flow cytometry and CyTOF data sets, on a single-processor machine with 8 GB of RAM.

Data Set	Number of Samples (FlowJo workspace files)	Size of data set	RAM usage (peak)	Number of Markers	Time to parse manual gates	Time to perform automated gating	Time to generate Boolean subsets
**HTN 080**	470 (16)	18.8 GB	4.7 MB (1.8 GB)	12	20.6 minutes	1.74 hours	2.6 minutes (7520 subsets)
**CyTOF**	3 (NA)	75 MB	8 K (886 MB)	21	manual gates unavailable	5 min	7.2 minutes. (2048 subsets)

OpenCyto can reproduce the FlowJo manual gates from a 16-workspace data set in 21 minutes with a peak memory usage of 1.8 GB. Once gated, the data occupies only 4.6 MB of RAM and is efficiently stored on disk in the HDF5/NetCDF format. Automated gating of the same data set using on OpenCyto GatingTemplate to generate data-driven gates for each of the 470 samples takes 1.74 hours on a single-processor. This can be parallelized across multiple cores for greater efficiency. The 420×2^4^ Boolean subsets of 4-cytokine producing cells can be generated and extracted efficiently, taking only 17 minutes for 7520 different subsets. Analogous results are shown for the CyTOF data, which has higher dimensionality. Calculating the Boolean subsets of 9 cytokine gates for the four maturation subsets in the data was extremely quick. In contrast, the 4×2^9^ Boolean subsets took 104 minutes to compute in FlowJo.

### OpenCyto Can Explore Cytokine Expression in CD8^+^ T Cell Subsets from CyTOF Data

**Figure 5:**
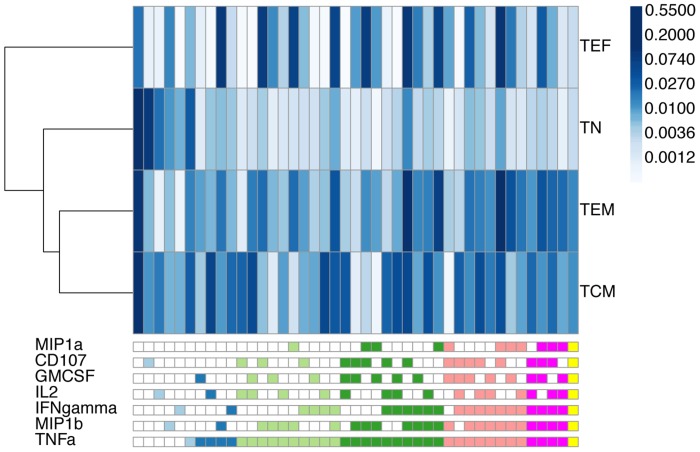
The average frequency of expression across two CyTOF samples for cytokine-producing cell subsets from four T-cell maturational states. Samples were stimulated with PMA-Ionomycin for 3 hours. Rows represent different maturational cell subsets (TN: naïve, TCM: central memory, TEF: effector, TEM: effector memory) and are clustered by Euclidean distance similarity. Columns represent different cytokine-producing cell subsets. The bottom legend defines the cell subset in a column. The legend is colored by degree of functionality of the cell subsets (light blue: degree 1, dark blue: degree 2, light green: degree 3, dark green: degree 4, salmon: degree 5, red: degree 6, orange: degree 7). The shading of individual blocks of the heatmap represents the average proportion of cells in the subset across the two samples, normalized to the total number of CD8 T-cells. Naïve cells have low polyfunctionality compared to effector, effector memory, and central memory cells.

**Figure 6:**
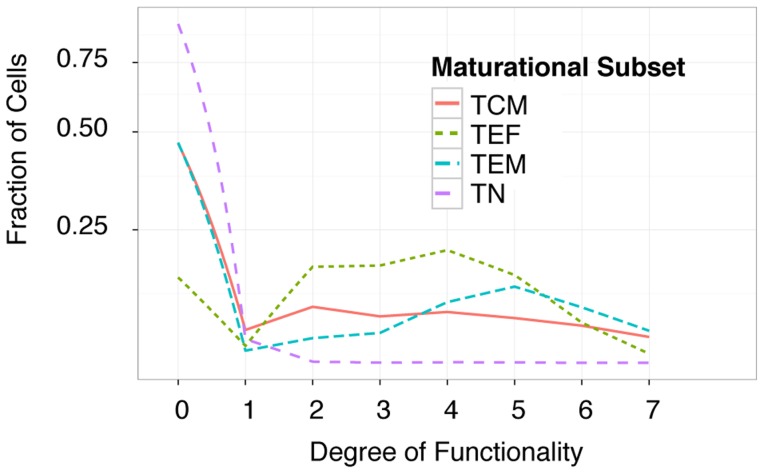
The distribution of cells of each maturational state and their degree of functionality. The majority of naïve CD8 T cells (TN) do not express any cytokines (degree of functionality 0) or are mono-functional, while effector memory cells (TEM) are the most polyfunctional of the subsets (peaking at degree 5). Short-lived effector (TEF) cells have lower polyfunctionality (peaking at degree 4), and central memory (TCM) populations tend to have a constant level of polyfunctionality from degree1 through degree 7. The area under the curve for each cell subset integrates to one. The y-axis is transformed by a hyperbolic-arcsine to facilitate visualization of differences between subsets at higher degrees of polyfunctionality.

Cytometry by time of flight was used to explore the expression of nine cytokine and functional markers on CD8^+^ T cells. The markers included TNF-α, IFN-γ, MIP1α, MIP1β, IL-2, GMCSF, CD107, Granzyme B, and perforin. In addition to these, the panel included markers used to identify naïve, short-lived effector, effector memory, and central memory T-cell maturational subsets. In total, twenty-three different markers or measurements of physical characteristics were used to identify individual events [30]. The thresholds for cytokine and functional marker positivity were derived from the non-stimulated sample and applied to the two stimulated samples presented in the figures of the original study [30]. This is a straightforward procedure within the OpenCyto framework (reproducible code can be found at opencyto.org). The complete gating hierarchy for the negative control and stimulated samples can be found in Figures S6 and S7, respectively. The same positivity threshold is used across samples and is based on the 99^th^ percentile of expression in the non-stimulated sample, as in the original publication [30]. The automated gating templates used to derive data-driven gates for the non-stimulated and stimulated samples are available in Files S3 and S4, and representative gates for non-stimulated and stimulated samples are shown in Figures S8 and S9, respectively. The data were filtered to remove cytokine producing cell subsets with less than 1% expression. This reduced the set of features to forty-three unique subsets of cytokine expressing cells across the 4 maturational states (Figure 5). In Figure 5 we show the average proportion of each cell subset across the two samples analyzed here, and observe clear differences across maturational states. We further summarized the expression in each maturational T-cell subset by computing the degree of functionality (polyfunctionality) of each set of cytokine producing cells and plotting their distributions (Figure 6). Naïve CD8 T-cells were observed to express zero, one or two cytokines, while short-lived effector CD8 T-cells were seen to have the highest degree of polyfunctionality, consistent with our understanding of the biology of these compartments (Figure 6).

Importantly, the analysis of the CyTOF data set demonstrates the flexibility of our framework, and how it can be extended to accommodate new types of data from new single-cell cytometric assays. For example, to analyze the CyTOF data set we implemented a new gate type (dnaGate) to identify “single-cells” in the DNA-DNA dimensions (Supporting Figures S8, S9 and Files S3 and S4). This is a non-standard gate that is the CyTOF equivalent of a singlet gate. Our plugin framework allows automated gating pipelines written in OpenCyto to be easily extended to leverage any of the automated gating or clustering algorithms available in the BioConductor ecosystem. This flexibility enables users to easily construct analyses specifically tailored to identify the cell populations of interest in their assays.

The hierarchical gating strategy, which is an explicit and integral part of the OpenCyto framework, is compatible with both classical manual analyses, as well as new, high-dimensional approaches (Figure S1 A–C). Importantly, by keeping track of the cell population hierarchy, the pipeline facilitates cell-population matching across samples, irrespective of which gating algorithm is used to identify specific cell subsets. This enabled us to identify and analyze all cytokine-producing cell subsets across the four T-cell maturational states in the CyTOF data without resorting to ad-hoc or heuristic cell population matching approaches. The framework even allows for missing populations. The cell hierarchy encodes important domain-specific knowledge about an experiment, which is preserved in our approach. As an example, the *gatingTemplate* for the ICS data set specifies the *PTID:VISITNO* experimental variables in the *groupBy* column of the template file for each cytokine gate (File S1). These correspond to the *subject* and *visit* associated with a specific FCS file, and instructs *OpenCyto* to *combine* these samples when gating cytokine channels, ensuring samples that need to be directly compared (i.e., stimulations and controls within a visit and subject) have a consistent gating threshold. This type of flexibility to combine and collapse samples can also be used to increase the density of cell subsets for very rare cell populations prior to gating, or to combine samples for Bayesian prior elicitation when using the *flowClust* gating method.

#### QA procedures and OpenCyto

Although OpenCyto does not have an explicit QA module, the standard QA procedures involving data visualization and exploration can readily be applied to the OpenCyto workflow. The *flowViz* package allows for flexible visualization of gates and cell populations, and R's statistical environment enables standard outlier detection methods to be applied to cell population statistics. A typical QA workflow in openCyto may involve iterative template development on a subset of a complete data set, with concomitant exploratory analysis of the results. Existing QA tools like *QUALIFIER* [26] are built around the same *flowWorkspace* framework and can also be used with OpenCyto *GatingSet* objects. Other tools like *FCSClean*/*FlowClean* can be integrated readily via the plugin framework [39]. The various *gatin*g *algorithm* tuning parameters are generally selected to provide gate thresholds that are subjectively appealing to the user, but are defensible on *objective* grounds (i.e. one can explain exactly *why* a given gating algorithm is selecting a certain cut-point, given the parameters). In the examples shown here, tuning parameters were selected with the idea in mind that the resulting gates are *not obviously wrong*, rather than being tuned to provide a good fit to manual gating. We would recommend such a strategy in general.

## Availability and Future Directions

While exhaustive documentation of the features of *OpenCyto* is beyond the scope of a manuscript, we have aimed to provide several use cases that demonstrate how the framework can be applied in practice. Further details, documentation, tutorials, and use case examples (including all code and data to reproduce the figures in this paper) are available online (http://www.opencyto.org), and the software can be downloaded from github (https://github.com/RGLab/openCyto). and from BioConductor (http://www.bioconductor.org).

The OpenCyto framework enables easy, automated, data-driven gating of high-dimensional (e.g., many samples or many dimensions) FCM data sets, eliminating the time-consuming task of manual gating. By incorporating expert-elicited and data-driven prior knowledge, OpenCyto attains accurate gating of cell populations, including rare populations, in an objective manner that is directly comparable to careful, expert manual gating. The ability to construct abstract, data-driven gating templates that incorporate any gating algorithm makes it a valuable tool for *core facilities* that frequently generate and analyze highly standardized data. The text-based gating template definitions lower the barrier to adoption of automated FCM data analysis methods by making the framework easier to use, minimizing the need to write data-set specific code and promoting reproducible data analysis that is easy to share. Similarly, built-in support for importing manual gates from external tools is designed to promote collaboration and facilitate the comparative analysis of the large quantities of existing flow data sets. Importantly, the *core* BioConductor flow packages already have a large user base and are widely used in a variety of fields [12], [16], [40]–[50]. The significant infrastructure improvements made to the *core* packages in order to support the OpenCyto framework will also greatly benefit this community. Future work will include further optimizations of the framework to improve speed, expansion of the repertoire of gating algorithms to include more CyTOF-specific methods, and development of a web-based graphical user-interface to further facilitate defining OpenCyto gating templates, as well as support for GatingML 2.0 compliant output (using *flowUtils*) of openCyto gates for bi-directional interoperability with FlowJo and better integration with cloud-based platforms like CytoBank [18].

## Supporting Information

Figure S1 Three examples using OpenCyto to perform automated gating using a hierarchical approach, a high-dimensional automated approach, and a hybrid approach. A) A hierarchical, pairwise gating scheme for identifying cytokine-producing T-cells. B) A naïve, high-dimensional approach to do the same as A, C) a hybrid approach combining pairwise hierarchical gating and high-dimensional gating of specific cell subpopulations from the hierarchical scheme. Different colored nodes represent cell populations identified via pairwise gating (light gray), high-dimensional gating (dark gray), or all events in the FCS file (white). Panel C) explicitly represents the approach undertaken by many high-dimensional automated gating algorithms.
(EPS)

Figure S2 Automated and manual gating hierarchy for HVTN 080. A) Hierarchy of automated gates and B) manual gates for HVTN080. Some additional filtering gates (boundary and debris event removal) were added to the automated gating scheme to clean up the data. The visualization was created using *flowWorkspace*.
(EPS)

Figure S3 Automated gating layout for a representative sample from data set HVTN 080. Data-driven gate thresholds were derived using openCyto and the gating template defined in File S1. The visualization was generated using the new functionality in the *flowViz* package.
(EPS)

Figure S4 Manual gates for the HVTN 080 data set imported from FlowJo. The layout shows the manual gates for a representative sample of the HVTN 080 data set. The gates were reproduced in openCyto from the FlowJo workspace using the *flowWorkspace* package. The visualization was generated using the *flowViz* package.
(EPS)

Figure S5 Paired difference of post-vaccine minus pre-vaccine proportions of POL-1-PTEG stimulated, background corrected, CD8^+^ and CD4+ T-cells expressing any cytokine AND perforin in HVTN080. There is a vaccine regiment specific trend for post-vaccine response in the CD8+ cell subset (one-sided simultaneous test of linear hypotheses, post-vaccine - pre-vaccine >0 within each vaccine regimen, based on a linear mixed effects model, with random subject effect, fit to the proportions). We observe more evidence for post-vaccine response in the CD8^+^ T-cell subset than the CD4^+^ subset, as expected. The POL-1-PTEG stimulation was chosen because it showed the largest response magnitude. Such an analysis is not possible with the manually gated data.
(EPS)

Figure S6 The hierarchy of automated gates for the negative control in the CyTOF data set. Thresholds for Perforin and Granzyme B were based on the spiked-in mouse lymphocytes expressing CD8.
(EPS)

Figure S7 The hierarchy of automated gates for the CyTOF data set. The visualization was created using *flowWorkspace*. The 2048 automatically generated Boolean gates are omitted for clarity. The thresholds for Perforin and Granzyme B were derived from the spiked-in mouse lymphocytes in the negative control sample.
(EPS)

Figure S8 Automated gating of an unstimulated sample from the CyTOF data set. The gating template in File S2 was applied to the unstimulated CyTOF sample to generate the data-driven gates shown here. The layout was generated using the new functionality in the *flowViz* package.
(EPS)

Figure S9 Automated gating of a stimulated sample from the CyTOF data set. The 2048 Boolean subsets automatically generated from the functional markers are not shown. Thresholds were taken from the gates derived from the non-stimulated sample, as in the original publication. The threshold for positivity of perforin and Granzyme B was defined using the spiked-in negative control mouse cells for CD8+ T-cells, as in the original publication.
(EPS)

File S1 OpenCyto gating template for the HVTN 080 study. Each row contains a cell population definition. Columns are provided for the population name (alias, pop), channels defining the population (dims), its relationship to other populations (parent), and the gating algorithm (gating_method) to be used to gate the population. Additional columns for each population are provided for algorithm-specific parameters (gating_args), and well as the ability to group (collapseDataForGating, groupBy) samples for gating and preprocessing based on sample-level metadata in the “phenoData” slot of the GatingSet object.
(XLSX)

File S2 OpenCyto gating template for gating the negative control in the CyTOF data set. The T-cell maturational subsets are not defined in this template, nor are the Boolean combinations of cytokine producing cells; only the marginal cytokine subsets are defined here, and used as reference gates for the stimulated samples.
(XLSX)

File S3 OpenCyto gating template for gating the stimulated samples from the CyTOF data set. The template contains definitions for T-cell effector, effector memory, central memory, and naïve cells as well as their cytokine producing subsets.
(XLS)

Text S1 Description of the statistical model used to identify cell subsets with antigen-specific changes induced upon vaccination in the HVTN 080 data set.
(DOCX)
