# Supplementary material for: OpenCyto: An Open Source Infrastructure for Scalable, Robust, Reproducible, and Automated, End-to-End Flow Cytometry Data Analysis
Source: PLoS Comput Biol. 2014 Aug 28;10(8):e1003806. doi: 10.1371/journal.pcbi.1003806 (PMC4148203; doi:10.1371/journal.pcbi.1003806)
Supplement: Text S1 — Description of the statistical model used to identify cell subsets with antigen-specific changes induced upon vaccination in the HVTN 080 data set. (DOCX) [file pcbi.1003806.s013.docx]

**Supplementary Information for “OpenCyto: An Open Source Infrastructure for Scalable, Robust, Reproducible, and Automated, End-to-End Flow Cytometry Data Analysis”**

**Authors**

Greg Finak^a^, Jacob Frelinger^a^, Wenxin Jiang^a^, Evan W. Newell^b^, John Ramey^a^, Mark M. Davis^c,d,e^, Spyros A. Kalams^f,g^, Stephen C. De Rosa^a,h^, and Raphael Gottardo^a,i,1^

**Affiliations**

^a^ Vaccine and Infectious Disease Division, Fred Hutchinson Cancer Research Center, Seattle, WA 98109, USA

^b^ Agency for Science Technology and Research, Singapore Immunology Network, Singapore 138648.

^c^ Department of Microbiology and Immunology, Stanford University, Stanford, CA 94305, USA

^d^ Institute for Immunity, Transplantation and Infection, Stanford University, Stanford, CA 94305, USA

^e^ The Howard Hughes Medical Institute, Stanford University, Stanford, CA 94305, USA

^f^ Infectious Diseases Division, Department of Medicine, Vanderbilt University School of Medicine, Nashville, TN **37232-2582**

^g^ Department of Pathology, Microbiology, and Immunology, Vanderbilt University School of Medicine, Nashville, TN **37232-2582**

^h^ Department of Laboratory Medicine, University of Washington, Seattle, WA 98195, USA

^i^ Department of Statistics, University of Washington, Seattle, WA 98195, USA

^1^ To whom correspondence should be addressed

**Supporting Text S1**

*Model Description*

In order to identify cell subsets that exhibit antigen-specific changes upon vaccination, we fit a linear mixed effects model to the background subtracted proportions of cytokine positive cells within each population and gating method. Without loss of generality, for a fixed cell subset and stimulation condition, we let

$y_{ijk}^{s} , y_{ijk}^{u}$ be the proportions of cytokine positive cells in the antigen stimulated ($s$) and non-stimulated ($u$) sample from subject$i$, visit $j$, and vaccine regimen $k$, and $y_{ijk}^{\Delta}=y_{ijk}^{s}-y_{ijk}^{u}$ be the background subtracted proportion of cytokine positive cells. We model:

$y_{ijk}^{\Delta}={\mu+ \gamma}_{i}+a_{j}+b_{k}+c_{jk}+\epsilon_{ijk}$,

where $\mu$ is the intercept for the cell subset,$\gamma_{i}$ is a subject-level random effect with $\gamma_{i}\sim N(0,\sigma_{I})$ , $a_{j}$ is the effect of visit $j$, $b_{k}$ is the effect of vaccine regimen $k$, and $c_{jk}$ is the interaction between visit $j$ and vaccine regimen $k$, and $\epsilon_{ijk}\sim N(0,\sigma_{\epsilon})$. We want to test whether $c_{jk}>0$ (i.e., whether there is an increase in the proportion of cytokine positive cells at the post-vaccine time-point compared to the pre-vaccine time-point) for each vaccine regimen. We fit the model with the R’s *lmer* function from the *lme4* package, and perform linear hypothesis tests using the *glht* and *contrasts* packages.
